# Estimating the public health impact had tobacco-free nicotine pouches been introduced into the US in 2000

**DOI:** 10.1186/s12889-022-13441-0

**Published:** 2022-05-21

**Authors:** Peter N. Lee, John S. Fry, Tryggve Ljung

**Affiliations:** 1 P.N.Lee Statistics and Computing Ltd., 17 Cedar Road, Sutton, Surrey SM2 5DA UK; 2 RoeLee Statistics Ltd., 17 Cedar Road, Sutton, Surrey SM2 5DA UK; 3 Swedish Match, Sveavägen 44 8th Floor, SE-118 85 Stockholm, Sweden

**Keywords:** Smoking, Public health, Modelling, Mortality, Nicotine pouches, USA

## Abstract

**Background:**

For smokers not intending to quit, switching to a reduced-risk nicotine product should be healthier than continuing smoking. We estimate the health impact, over the period 2000–2050, had the nicotine pouch ZYN hypothetically been introduced into the US in 2000. ZYN’s toxicant profile and method of use is like that for Swedish snus, a product with known health effects much less than smoking.

**Methods:**

Our modelling approach is similar to others developed for estimating potential effects of new tobacco products. It starts with a simulated cohort of 100,000 individuals in the year 2000 subdivided by age, sex, and smoking status (including years since quitting). They are followed annually accounting for births, net immigrations, deaths and product use changes, with follow-up carried out in the Base Case (ZYN not introduced) and Modified Case (ZYN introduced). Using informed assumptions about initiation, quitting and switching rates, distributions of the population over time are then constructed for each Case, and used to estimate product mortality based on assumptions about the relative risk according to product use.

**Results:**

Whereas in both Base and Modified Cases, the prevalence of any current product use is predicted to decline from about 22% to 10% during follow-up, in the Modified Case about 25% of current users use ZYN by 2050, about a quarter being dual users and the rest ZYN-only users. Over the 50 years, deaths at ages 35–84 from product use among the 100,000 are estimated as 249 less in the Modified than the Base Case, equivalent to about 700,000 less in the whole US. Sensitivity analyses varying individual parameter values confirm the benefits of switching to ZYN, which increase as either the switching rate to ZYN increases or the initiation rate of ZYN relative to smoking increases. Even assuming the reduction in excess mortality risk using ZYN use is 20% of that from smoking rather than the 3.5% assumed in the main analyses, the reduction in product-related deaths would still be 213, or about 600,000 in the US.

**Conclusions:**

Although such model-based estimates involve uncertainties, the results suggest that introducing ZYN could substantially reduce product-related deaths.

**Supplementary Information:**

The online version contains supplementary material available at 10.1186/s12889-022-13441-0.

## Background

Smoking is a major cause of preventable disease, and responsible for many millions of deaths and years of life lost [1]. While the healthiest option for smokers is to quit smoking immediately and completely, many public health experts and institutions have embraced harm reduction as an alternative approach in tobacco control [2–4]. Tobacco harm reduction is aimed at adult smokers who would otherwise continue smoking and attempts to persuade them to switch to smoke-free alternatives such as snus, e-cigarettes or heated tobacco products; all of which have been shown to substantially reduce toxicant levels as compared to cigarettes [5–8].

Evidence to support tobacco harm reduction comes from extensive epidemiological findings over many years relating to Swedish smokers switching to snus. Studies clearly show that any increase in disease and death rates associated with the use of snus is much less than that associated with smoking [5, 9]. In Sweden, where snus use is common, the prevalence of smoking and the incidence of mortality from diseases related to nicotine and tobacco are the lowest in Europe [10].

Swedish Match recently introduced a new product known as ZYN that is currently marketed in the US and Europe. ZYN is a non-heated, tobacco-free, smoke-free and spit-free nicotine pouch for oral use with an appearance similar to Swedish snus products. Use of ZYN does not involve any inhalation of smoke or vapour. It is intended for adult tobacco and nicotine consumers, and comes in different flavours and nicotine strengths. It is intended to be used under the upper lip for up to an hour and then discarded. Chemical characterization of nicotine pouches and snus suggests that users of nicotine pouches are exposed to lower levels of toxic compounds than are users of snus [11].

For products introduced more recently into the market various modelling approaches have been used to estimate their future population health impact. These techniques are based on various informed assumptions, including their rate of uptake, the effect their uptake has on the prevalence of cigarette smoking, and the relative toxicity of the product compared to cigarettes. A review of these approaches has recently been published [12]. Some approaches concern specific new products, such as e-cigarettes or heated tobacco products, while others are more general, using terms like modified risk tobacco product, new nicotine product, or new tobacco product [12].

Here we use a dynamic population microsimulation model to estimate the effect that the hypothetical introduction of ZYN in 2000 might have had in the US on overall mortality and on the combined distribution of smoking and ZYN use over the subsequent 50 years. The approach used has similarities to that of Vugrin et al. (2015) [13] and has also been used by Apelberg et al. (2018) [14] when studying the effects of reducing nicotine levels in cigarettes from 2016 in the US. These similarities include the methodology used to correct for changes in mortality over time, the assumption that product-related death does not occur in those aged less than 35 years, and the data sources used to determine the initial cigarette smoking distribution, the distribution of time quit for former smokers, never smoker death rates above age 34 years, and the relative risk for smoking compared to never having smoked.

While a number of other modelling approaches concern follow-up from a recent year to the distant future [12], the “hindcasting” approach, involving determining what would have happened had a new product been introduced in the past, has also been frequently used [13, 15–18]. Advantages of the hindcasting approach include there being real world data for calibrating the Base Case, and the avoidance of extrapolating far into the future, with consequent increased uncertainty on future trends in tobacco use and on the effects on future mortality rates of exogenous factors, such as medical progress and infections.

## Methods

### Outline of the approach

#### The approach involves three stages

The first stage defines the population in the baseline year, 2000. A hypothetical population of 100,000 US individuals is subdivided by sex and age, and also into never, current and former cigarette smokers, with former smokers subdivided by years quit. The new product, ZYN, is assumed not to have been available before 2000.

In the second stage, the population is followed up annually until 2050. Every year the population is updated to take into account births, net immigrations, deaths and changes in nicotine product use, with the distribution of sex, age and cigarette smoking in the immigrant population assumed to be equal to that used for the initial population.

Follow-up is carried out in two scenarios: the “Base Case” where the new product, ZYN, is never introduced; and the “Modified Case” where it is introduced immediately after baseline. Note that the use of nicotine-containing products other than cigarettes or ZYN is not considered in either scenario.

Table 1 details the nicotine use groups considered. In the Base Case, which concerns only groups 1 (never smokers), 2 (current cigarette smokers) and 3 (former cigarette smokers), our application of the model allows individuals to initiate smoking (change from group 1 to 2) or to quit smoking (change from group 2 to 3), but not to re-initiate smoking (change from group 3 to 2). Transitions to never smokers (group 1) are not possible, while transitioning from never to former smokers (change from group 1 to 3) is assumed not to occur in a single period.

**Table 1:** Nicotine use groups

	Cigarette use		
ZYN use	Never	Current	Former
Never	1	2	3
Current	4	5	6
Former	7	8	9

In the Modified Case nine groups are involved:

- 1–3: never ZYN by never, current and former cigarettes;
- 4–6: current ZYN by never, current and former cigarettes and
- 7–9: former ZYN by never, current and former cigarettes.

Twelve transitions between the groups are considered. Three relate to initiation (changes from group 1 to each of groups 2, 4 and 5), three to quitting (changes from group 2 to 8, 4 to 6 and 5 to 9) and six to switching (changes from group 2 to 5, 2 to 6, 4 to 5, 4 to 8, 5 to 6 and 5 to 8). Other possible transitions are not considered, for comparability with the Base Case.

Based on the initial population distribution, the transition probabilities between groups, and changes in the simulated population due to births, net immigrations and deaths, data are available at each year from 2000 to 2050 on the distribution of the population by age, sex and nicotine use for both the populations in the Base Case and the Modified Case.

Finally, in the third stage, the data on the distributions by age, sex and nicotine use are processed to estimate mortality associated with nicotine use from all causes, the difference between the two measuring the population health impact of introducing ZYN.

Additional details of the methods and data used are described below.

### Population at baseline at year 2000

The distribution of age and sex for the US population in 2000 was derived from the Human Mortality Database [19], which in turn used the 2000–2010 intercensal datasets published by the US Census Bureau [20]. 49.1% of the population was male and 50.9% female. The distribution by age group within sex is given in Additional File 1 Table A1.1.

The initial cigarette smoking distribution by sex and age group is as used by Vugrin et al. [13], based on data from the National Health Interview Survey (NHIS), and is given in Additional File 1 Table A1.2. The NHIS data only apply to age 18 or over. For ages below 18, the Cancer Intervention and Surveillance Modelling Network (CISNET) provide the current smoking data by age shown in Additional File Table A1.3. CISNET gives no information on former smoking, and the modelling assumes that there are no former smokers age 17 or less in the initial population.

Additional File 1 Table A1.4 gives, by sex and age group, the distribution of years quit in former smokers aged 18 or over, again as used by Vugrin et al. (2015) [13] based on NHIS data.

### Net migration and births

At every year during follow-up, the population is scaled up to allow for the inclusion of immigrants and new births. The sex distribution in newborns and the sex, age and cigarette use distributions for the immigrants are both assumed to be the same as at baseline. The estimates of net migrations and births used were the US Census Bureau 2008 National Population Projections, and are shown in Additional File 2 Tables A2.1 (migrations) and A2.2 (births).

### Estimation of histories of cigarette smoking for the Base Case

Additional File 3 Table A3.1 gives the smoking initiation and cessation rates by sex and age used in the Base Case. Full details of the methodology are also given in Additional File 3. In summary, using methodology similar to that of Vugrin et al. (2015) [13], these rates were derived from smoking histories for birth cohorts reconstructed from NHIS data. Initiation rates for ages 0–19 were derived from the 1980–1984 cohorts, for ages 20–24 from the 1975–1979 cohorts and so on. Initiation rates for individuals above age 30 are set to zero, as nearly all initiation occurs at younger ages [21]. Derivation of cessation rates was similar, with rates for ages 0–18 from the 1980–84 cohorts, for ages 19–24 from the 1975–79 cohorts, and so on. CISNET rates were available up to age 84, older individuals being assigned the rate for age 84. As cessation rates in the CISNET data represent successful smoking cessation rates for at least two years, re-initiation rates are set to zero. Both the initiation and cessation rates are assumed to be time independent.

### Estimation of histories of product use for the Modified Case

In the pre-approval market setting, transition probabilities in the Modified Case are necessarily based on assumptions that cannot be validated. The transition probabilities in the Modified Case were derived from those in the Base Case as follows:

#### Initiation

Overall initiation rates were set equal to that for initiating smoking in the Base Case, but split in the proportion 70:25:5 for initiation to exclusive cigarette smoking, exclusive ZYN use, and dual use.

#### Quitting

Each of the three quitting rates, from exclusive use of either product or of dual use were set equal to that for quitting smoking in the Base Case.

#### Re-initiation

All re-initiation rates were set to be zero.

#### Switching from current exclusive smoking to current exclusive ZYN use

Switching rates were taken as 0.18%, 0.24%, 0.36% and 0.54% for the age groups 10–14, 15–19, 20–24 and 25 + respectively.

#### Switching from current exclusive smoking to current dual use

Switching rates were taken as 0.04%, 0.05%, 0.07% and 0.11% for the age groups 10–14, 15–19, 20–24 and 25 + respectively.

#### Other switching rates

These were taken as 0.48% for age 10 + .

In the absence of other information, all the switching rates were as used in a previous publication [15] concerning the health impact of introducing a reduced risk tobacco product into the US. However, the initiation and quitting rates were chosen merely to be values that seemed not implausible. Although all these rates have considerable uncertainty, sensitivity analyses were also conducted (see below) so that the effect of using alternative estimates for the rates could be studied.

### Estimating mortality related to cigarette smoking in the Base Case

US death rates for never smokers aged 0–34 in 2000, given in Additional File 4 Table A4.1, were extracted from the Human Mortality Database [19]. As smoking-related mortality is considered minimal before age 35 [22], the risk of death for current smokers was assumed to be equal to that for never smokers, an assumption also made by Vugrin et al. (2015) [13].

Death rates for never and current smokers for ages 35 and above are taken from Vugrin et al. (2015) [13], the never smoker death rates being estimated from NHIS Linked Mortality Files. Similarly to Vugrin et al. (2015) [13], the never smoker death rates are adjusted for changes over the period 2000–2050 using mortality scaling factors estimated using the Lee-Carter method [23], and are available in Additional File Table A4.2 (males) and Table A4.3 (females). Individuals aged 85 years or older were assumed to face the same risk of death, regardless of age, given the same sex and smoking history. Individuals were tracked until age 101, being then counted as dead. The never smoker death rates for ages 35 and above were then converted into annual probabilities of death by sex and age groups using standard demographic methods [13], and are shown in Additional File 4 Table A4.4.

Current smoker death rates were calculated by multiplying the never smoker death rates by the estimates of relative risk by age and sex given by Vugrin et al. (2015) [13], which were derived from NHIS Linked Mortality Files, and are shown in Additional File 4 Table A4.5.

Former smoker probabilities were estimated, following Hill and Camacho (2017) [24], by assuming that the decline in excess risk (compared to never smokers) follows a negative exponential function, with a half-life of 9.08 years. Thus a current smoker with an excess risk of E at the time of quitting, would have an excess risk of E/2 at 9.08 years after quitting, and of E/4 at 18.16 years after.

### Estimating mortality related to cigarette smoking and ZYN use in the Modified Case

The risk of death for those who have never used cigarettes or ZYN was taken to be that of never smokers in the Base Case. The excess risk of exclusive current ZYN users was taken to be 3.5% of that of exclusive current smokers of the same sex and age, while the excess risk of current dual users was taken to be that of current smokers. The estimate of 3.5% was based on the average of estimates made of 5% for snus use and 2% for nicotine replacement products made by a panel of experts at the Independent Scientific Committee on Drugs [25]. For current ZYN users who were former smokers, the excess risk was taken to be the maximum of that for current ZYN use and former smoking. For former ZYN users who were current smokers, the excess risk was taken to be that of current smokers. For former ZYN users who had never smoked, the excess risk for current ZYN users was scaled down using the same half-life as used for former smokers. For former users of both products, the excess risk was taken to be the maximum of that for former ZYN use and former smoking.

Note that, in the following text, the term “product-related deaths” is used generally to describe either deaths related to cigarette smoking in the Base Case or deaths related to both cigarettes and ZYN in the Modified Case.

### Sensitivity analyses

Sensitivity analyses investigated the effect of varying the assumed values of each of a number of different parameters:

#### Excess risk factor for exclusive ZYN use

Instead of 3.5%, alternatives of 0%, 7% and 20% were tested.

#### Excess risk factor for dual use

Instead of taking the maximum of the individual excess risks from smoking and from ZYN use, alternatives of the mean and the sum were tested.

#### Excess risks for current smoking

Instead of using the excess risks implied by the relative risk values shown in Additional File 4 Table A4.5, the excess risks were multiplied either by 0.8 or by 1.2.

#### Half-life of the negative exponential function

Instead of using 9.08 years, values of 4.54 or 18.16 years were used,

#### Transition probabilities

For each of the quitting rates and the switching rates, alternatives of half or double the rate used in the Modified Case were used. For initiation, the Modified Case rates were constrained to add to the initiation rate used in the Base Case. One set of sensitivity analyses for initiation retained this constraint, so that if one of the rates were halved or doubled, the three rates were then multiplied by a scaling factor so that their sum remained the same. The other set of sensitivity analyses, which varied only the initiation rates for exclusive ZYN use and for dual use, did not keep this constraint, but kept the initiation rate to cigarettes unchanged, so allowing study of the possibility that use of ZYN might affect overall initiation rates.

Note that most of the sensitivity analyses only affect the Modified Case. However, the sensitivity analyses which vary the excess risks for current smoking, the half-life of the negative exponential function, or the quitting rate for cigarette smoking, affect both the Base and the Modified Case.

Each of the sensitivity analyses described above varies only one parameter at a time. A further analysis was carried out based on a Pessimistic Scenario in which 12 assumptions relating to the introduction of ZYN were varied simultaneously, each of which were found to reduce the advantage to ZYN when considered individually. Thus the excess risk for exclusive ZYN was taken to be 20% of that from smoking, and the excess risk for dual use was taken to be the sum of those for smoking and ZYN individually. Also the transition probabilities were halved for initiation rate for ZYN, quitting rates for ZYN and for dual use, and switching rates from smoking to ZYN use, from smoking to dual use, and from dual use to ZYN, while the transition probabilities were doubled for initiation rate for dual use, and switching rates from ZYN to smoking, from dual use to smoking, and from dual use to ZYN. The changes to the initiation rates described were made while keeping the overall initiation rate fixed.

## Results

Table 2 shows the predicted prevalences of tobacco use in the Base Case and the Modified Case by 10 year period over the 50 year follow-up period. For the sexes combined, the prevalences of never and former cigarette smokers are very similar in the Base and Modified Cases, never smokers rising steadily from almost 50% at baseline to over 63% after 50 years, and former smokers rising slightly from about 28% at baseline to about 30% at 2020, then falling to about 27% at 2050. For the Base Case the prevalences of current cigarette smokers falls steadily from about 22% at baseline to about 10% after 50 years, while for the Modified Case the prevalence of current cigarette-only smokers declines more to about 7%, but the prevalence of ZYN users (alone or with cigarettes) increases from zero at baseline to 2.5% after 50 years.

**Table 2:** Predicted prevalence of tobacco use in the Base and Modified Case

			Year					
Sex	Case	Tobacco use	2000	2010	2020	2030	2040	2050
Males	Base	Never cigarettes	42.3	47.9	52.8	57.0	59.9	61.6
		Former cigarettes	32.8	33.6	33.1	31.4	29.6	28.1
		Current cigarettes	25.0	18.5	14.1	11.6	10.5	10.3
	Modified	Never any product	42.3	47.9	52.8	57.0	59.9	61.6
		Former cigarettes	32.8	33.6	33.1	31.4	29.6	28.1
		Current cigarettes only	25.0	18.1	13.2	9.9	8.3	7.8
		Current ZYN only	0.0	0.3	0.7	1.3	1.6	1.9
		Current dual use	0.0	0.1	0.2	0.4	0.5	0.6
Females	Base	Never cigarettes	56.2	58.6	60.5	62.2	64.0	64.9
		Former cigarettes	23.9	26.4	27.9	27.7	26.7	25.6
		Current cigarettes	19.9	15.0	11.6	10.1	9.3	9.4
	Modified	Never any product	56.2	58.6	60.5	62.2	64.0	65.0
		Former cigarettes	23.9	26.4	27.9	27.7	26.7	25.5
		Current cigarettes only	19.9	14.6	11.0	8.6	7.3	7.0
		Current ZYN only	0.0	0.3	0.5	1.1	1.5	1.8
		Current dual use	0.0	0.1	0.1	0.4	0.5	0.6
Combined	Base	Never cigarettes	49.6	53.5	56.8	59.7	62.0	63.3
		Former cigarettes	28.1	29.9	30.4	29.5	28.1	26.9
		Current cigarettes	22.3	16.7	12.8	10.8	9.9	9.8
	Modified	Never any product	49.6	53.5	56.7	59.7	62.0	63.3
		Former cigarettes	28.1	29.9	30.4	29.5	28.1	26.8
		Current cigarettes only	22.3	16.3	12.1	9.3	7.8	7.4
		Current ZYN only	0.0	0.3	0.6	1.2	1.6	1.9
		Current dual use	0.0	0.1	0.2	0.4	0.5	0.6

Note that in the Modified case, the prevalences for former cigarettes include those who have used ZYN

At baseline, females compared to males have a smaller prevalence of former and current cigarette smokers, but by the end of follow-up these differences decline, in both the Base Case and the Modified Case. By the end of the follow-up the prevalences of current smoking (cigarettes, ZYN only, or dual) are quite similar in males and females.

Although the predicted uptake of ZYN in the Modified Case by 2050 is only 2.5%, reductions in overall deaths and deaths due to the products are clearly evident, as seen in Table 3, which presents cumulative results for ages 35 to 84 years based on the population considered (100,000 initiating in 2000). Overall, there is an estimated reduction of 113 total deaths and 249 product-related deaths, the smaller difference for total deaths reflecting the fact that some of those who do not die of smoking-related diseases due to the introduction of ZYN will instead die later, but still before age 85 years, of other diseases not related to smoking. Given that the US population in 2000 was about 282 million, one would have to multiply the estimated reductions by about 2820 to make them relevant to the whole population. When scaled up in this manner the reduction in numbers due to introduction of ZYN is about 320,000 total deaths and about 700,000 product-related deaths.

**Table 3:** Cumulative deaths at ages 35–84 in the Base Case and Modified Case

			Year					
Sex	Endpoint	Case	2000	2010	2020	2030	2040	2050
Males	Deaths due to product(s)	Base	0	1440	2852	4342	5837	7231
		Modified	0	1439	2845	4317	5778	7123
		Difference	0	1	8	25	59	109
	All deaths	Base	0	2970	6109	9678	13,526	17,260
		Modified	0	2969	6100	9659	13,490	17,194
		Difference	0	1	9	19	36	66
Females	Deaths due to product(s)	Base	0	1229	2423	3651	4934	6070
		Modified	0	1228	2416	3621	4857	5936
		Difference	0	1	7	30	77	134
	All deaths	Base	0	2622	5387	8367	11,616	14,563
		Modified	0	2622	5382	8354	11,591	14,516
		Difference	0	0	5	13	25	53
Combined	Deaths due to product(s)	Base	0	2665	5267	7982	10,762	13,299
		Modified	0	2663	5252	7925	10,623	13,049
		Difference	0	2	15	57	139	249
	All deaths	Base	0	5592	11,496	18,045	25,142	31,823
		Modified	0	5591	11,482	18,013	25,081	31,710
		Difference	0	1	14	32	61	113

Table 4 summarizes results from the Modified Case and 37 sensitivity analysis variations, giving the reductions as compared to the Base Case in product-related deaths and increases in life-years, both calculated for age 35 to 84 years. For an individual, life-years is calculated by the total number of years spent in this age-group during follow-up. It should be noted that while most sensitivity analyses affect only the outcome in the Modified Case where ZYN is introduced, in some analyses (7–10, 21, 22) both the Base Case and the Modified Case are affected.

**Table 4:** Differences between the Modified and Base Case after 50 year follow-up: sensitivity analyses

Analysis	Parameter varied	Variation	Reductions in product-related deaths at age 35–84 years	Increases in life-years aged 35–84
1	None – Main analysis		249.0	1419
2	Excess risk for exclusive ZYN^a^	0%	255.5	1434
3		7%	242.2	1372
4		20%	213.4	1230
5	Excess risk for dual use^b^	Mean	291.8	1678
6		Sum	244.7	1339
7	Excess risk for current smoking	× 0.8	179.4	1060
8		× 1.2	311.4	1857
9	Half-life (years)	4.54	235.5	1687
10		18.16	273.2	1517
	Initiation rates compared to base case for smoking: ZYN: dual^c^			
	(a) *overall initiation rate fixed*			
11	Halve smoking initiation rate	35: 54.2: 10.8	462.4	2225
12	Double smoking initiation rate	82.4: 14.7: 2.9	166.3	1094
13	Halve ZYN initiation rate	80: 14.3: 5.7	165.2	1075
14	Double ZYN initiation rate	56: 40: 4	355.7	1830
15	Halve dual initiation rate	71.8: 25.6: 2.6	246.2	1341
16	Double dual initiation rate	66.7: 23.8: 9.5	238.0	1333
	(b) *overall initiation rate varies*			
17	Halve ZYN initiation rate	70: 12.5: 5	345.5	1504
18	Halve dual initiation rate	70: 25: 2.5	272.6	1308
19	Double ZYN initiation rate	70: 50: 5	26.7	1405
20	Double dual initiation rate	70: 25: 10	161.9	1167
21	Quitting rate for smoking	× 0.5	353.1	2060
22		× 2	138.5	709
23	Quitting rate for ZYN use	× 0.5	235.9	1467
24		× 2	273.0	1596
25	Quitting rate for dual use	× 0.5	234.6	1359
26		× 2	267.8	1451
27	Switching rate from smoking to ZYN	× 0.5	220.5	1126
28		× 2	311.0	2239
29	Switching rate from ZYN to smoking	× 0.5	263.1	1529
30		× 2	226.0	1296
31	Switching rate from smoking to dual	× 0.5	247.7	1419
32		× 2	252.1	1432
33	Switching rate from dual to smoking	× 0.5	248.9	1419
34		× 2	248.2	1404
35	Switching rate from ZYN to dual	× 0.5	264.1	1505
36		× 2	222.6	1232
37	Switching rate from dual to ZYN	× 0.5	247.7	1419
38		× 2	252.1	1432
	Pessimistic Scenario^d^		19.9	237

In analyses 1–6, 11–20 and 23–38 the base case is the same, and the cumulative numbers of product-related deaths and life-years are 13,298.5 and 3,246,177. In other analyses the variation affects both the base and modified case. For analyses 7–10, 21 and 22 the product-related deaths in the base case are, respectively, 11,735.2, 14,741.6, 12,165.2, 14,979.1, 14,224.4 and 12,240.8, while the life-years are 3,268,987, 3,223,069, 3,262,130, 3,223,202, 3,236,449 and 3,258,176

^a^Excess risk is 3.5% for modified case

^b^Excess risk is maximum of the risks for cigarettes and for ZYN in the modified case

^c^The first six sets of analyses (11–16) fix the overall initiation rate, while halving or doubling one of the rates, while the next four sets (17–20) have no such restriction

^d^This simultaneously makes each of the changes in analysis 4, 6, 13, 16, 23, 25, 27, 30, 31, 34, 36 and 37

In all the sensitivity analyses, there is a decrease in product-related deaths. The smallest decrease is in analysis 19, where the initiation rate of ZYN is doubled with no constraint on the overall initiation rate, so that the overall initiation rate for cigarettes and ZYN combined is increased by 25%. Here the extra overall initiation rate is more than compensated for by the fact that, in the Modified Case, cigarette smokers can switch to ZYN. The largest changes in product-related deaths between the range of parameter values being compared generally relates to cigarette smoking. Thus, there are differences of 296.1 (462.4 vs 166.3) according to whether the smoking initiation is halved or doubled (analyses 11 and 12 where the overall initiation rate is constrained), 214.6 (353.1 vs 138.5) according to whether the quitting rate for smoking is halved or doubled (analyses 21 and 22 where the variation applies both to the Base and Modified Cases), and 132.0 (311.4 vs 179.4) according to whether the excess risk for current smoking is multiplied by 1.2 or 0.8 (analyses 7 and 8). However, there is also a large difference of 190.6 (355.7 vs 165.2) according to whether the initiation rate of ZYN is doubled or halved (analysis 13 and 14 where the overall initiation rate is constrained). Given the uptake of ZYN is assumed to be quite low in our Modified Case (see Table 2) it is possible that initiation rates of ZYN might be even higher than this, with a consequently larger reduction in product-related deaths. There is also a reasonably large difference of 90.5 (311 vs 220.5) in relation to whether rates of switching from smoking to ZYN are doubled or halved.

It is also clear from Table 4 that in a number of the pairs of analyses related to dual use (15 and 16, 31 and 32, 33 and 34) there are only small changes in product-related deaths between the pairs of parameter values compared. In a number of other sets of analyses (2 to 4, 5 and 6, 9 and 10, 23 and 24, 29 and 30) a difference in product-related deaths between the parameter values tested is evident, but of smaller magnitude, typically about 40, variations in these parameters not having a major effect on the conclusion from the main analysis (analysis 1).

A number of the conclusions from the sensitivity results for the increases in life years and those for the decreases in product-related deaths are similar. Thus, there are consistent increases in life-years as there were decreases in product-related deaths in each analysis. Also, as before, the variation in the estimates is largest for those where the changes involve cigarette smoking (compare analyses 8 and 7 for excess risk, 12 and 11 for initiation, 22 and 21 for quitting, and also 6 and 5 for dual use), initiating with ZYN (compare analyses 14 and 13) and switching from smoking to ZYN (compare analyses 27 and 28). However, some differences must be noted. Firstly, while generally changes in parameter values that increase product-related deaths also decrease life-years (and vice versa), the changes following variations in the half-life estimate (see analyses 9 and 10) do not follow this general pattern. Here reductions in product-related deaths increase from 235.5 to 273.2 as the half-life estimate changes from 4.54 to 18.16 but increases in life-years reduce from 1687 to 1517.

Second, if one expresses the estimates as ratios from the main analysis, the values are very similar for the two impact measures, but this is not always true. Thus, while the ratios (details not shown) are quite similar in the analysis of the excess risk for current smoking for analyses 7 and 8 (0.72 and 1.25 for deaths, and 0.75 and 1.31 for life-years), and are also similar for analyses 2–6, 15–16, 21–26 and 29–38) they are not very similar in some other cases, apart from for life-years. These include analyses 11 and 12 (smoking initiation rate), 13 and 14, and 17 and 19 (ZYN initiation rate), 18 and 20 (dual initiation rate), and 27 and 28 (switching rate from smoking to ZYN). In each case, the variations in parameter values have more proportional effect on deaths than life-years. The most striking case of this is seen in analysis 19, where the beneficial impact of introducing ZYN almost disappeared as regards deaths, but not as regards life-years.

Table 4 also includes results from the Pessimistic Scenario, which simultaneously made each of the changes described in analyses 4, 6, 13, 16, 23, 25, 27, 30, 31, 34, 36 and 37, an advantage was still seen resulting from the introduction of ZYN. Here there was an estimated reduction in product-related deaths of 20 and increase in life-years of 237 as compared to the Base Case. These compare with the estimates of 249 and 1419 in our main analysis, the Modified Case.

## Discussion

Based on modelling of the effects of introducing ZYN into a sample of 100,000 of the US population in 2000, with subsequent follow-up until the year 2050, our main analysis estimates that tobacco-related deaths would reduce by 249 as compared to the Base Case where ZYN is not introduced. This is equivalent to about 700,000 deaths in the whole US population. These estimates are based on product transition rates which suggest that, by the year 2050, about 25% of current total users of cigarettes or ZYN will be using ZYN. Sensitivity analyses suggest that a substantial reduction of tobacco-related deaths following the introduction of ZYN would still be evident unless the assumed initiation rate of ZYN was considerably increased, with no concomitant reduction in the assumed initiation rate of cigarette smoking.

Based on the assumptions described in the Methods section we predict that, in 2050, the proportion of current users in the Base and the Modified Case will be the same. This does not seem inconsistent with data on trends in e-cigarettes in the US [26] and on trends in snus use in Norway [27] where much larger switches away from cigarettes to the alternative product have been observed.

Various modelling approaches have been used to estimate the population health impact of introducing modified risk tobacco products [12]. The one used here is similar to that used by Vugrin et al. (2015) [13] who have discussed its strengths and weaknesses. The strengths include the use of population and mortality projections which closely correspond to Census projections, and the use of smoking initiation and cessation rates from recent cohorts, so that smoking prevalence projections fit in with observed US estimates based on NHIS data. Limitations mentioned by Vugrin et al. (2015) [13] include the restriction to all-cause mortality, while some other modelling approaches consider the major smoking-related causes of death individually [15], using a modelling approach which does not take into account daily cigarette consumption approach or years smoked, and assuming that smoking initiation and cessation rates remain constant over time.

It should also be noted that the modelling fails to take into account pipe and cigar smoking, smokeless tobacco, use of modified risk tobacco products and environmental tobacco smoke. While these factors have been considered unlikely to have a material effect on the results in other modelling approaches [15], this failure is nevertheless a limitation, as it may affect assumptions about the uptake and initiation of ZYN.

The complexity of taking other products fully into account is emphasized by a very recent study [28] of 1305 US residents aged 21 + who had used ZYN at least once per week in the previous 30 days. Of those, the numbers (percentages) reporting every day or some days use of other products was 666 (51.0%) for other nicotine pouches, 254 (19.5%) for moist snuff, 241 (18.5%) for e-cigarettes, 202 (15.5%) for snus, 196 (15.0%) for cigars/cigarillos, 187 (14.3%) for cigarettes, 88 (6.7%) for chewing tobacco, 67 for other products, 47 (3.6%) for pipes, and 29 (2.2%) for hookah/water pipe, with only 336 (25.7%) being exclusive ZYN users. Use prior to first ZYN use was also studied, with the number (percentage) for other nicotine pouches 546 (41.8%), and those for all the other products about doubled including cigarettes, 382 (29.3%). It would clearly take very detailed data, not currently available, on prevalence of and transitions between the 11 smoking groups and relative risks by product to carry out a fuller analysis.

An alternative approach to estimating tobacco transition probabilities would have been to derive them based on published estimates for smokeless tobacco in the US [29, 30] as a proxy. We did not use this approach for two reasons. First, the purpose of our article was to model the public health effect of introducing a novel product on the market, not to model the effect of an improved smokeless product. Second, actual sales data suggest that ZYN has received a different reception from consumers compared to traditional smokeless tobacco, with traditional smokeless tobacco sales remaining stable or even somewhat declining [31], whereas the sales of ZYN have grown [32]. In any case, our detailed sensitivity analyses give good insight into the public health impact of variation in the assumed values of the transition probabilities.

Another limitation of our modelling is that, though the estimated transition probabilities between smoking groups for the Base Case vary by sex, the assumptions used to derive from these the transition probabilities for the Modified Case are assumed to be independent of sex. For example, the proportion of men and women who initiate with ZYN among those who, in the Base Case would initiate with smoking, may indeed vary by sex, and indeed the study cited above [28] of 1.305 ZYN users included 88% males. In the absence of reliable data, we used a simplistic approach, which will probably overestimate reductions in mortality in one sex, and underestimate the reductions in the other sex.

Our modelling approach assumes that there is no re-initiation of smoking by those who have quit, since the derived cessation rates represent successful cessation rates for at least two years. While this follows Vugrin et al. (2015) [13], one should note that our estimates may be optimistic if those who have quit smoking try ZYN, and because of this are led to revert to smoking.

Our estimates ignore the possibility that changes over time may occur that have nothing to do with tobacco use. These may include, for example, effects of medical advances, new infections, wars and global warming. But such factors are typically not considered in modelling, even in approaches which consider deaths occurring up to the year 2100, e.g. [14, 33].

As with all such modelling approaches, the scenarios examined are hypothetical, and the results depend on the assumptions made. To illustrate the effect of uncertainties relating to the different assumptions, we include the results of various sensitivity tests. Thus, while our Modified Case assumes that the excess mortality risk of ZYN is only 3.5% of that from cigarettes, consistent with extensive evidence from epidemiological studies on snus [5, 9], we also report the results of analyses in which this percentage can rise to 20%. Similarly we test for variations in the assumed values of the excess risk factor for dual use, the relative risk for current smoking and the quitting half-life, as well as variations in the assumed initiation, quitting and switching rates. Generally the results from these sensitivity analyses confirm that the introduction of ZYN into the US population would cause a substantial reduction in product-related deaths.

## Conclusions

Had ZYN been introduced into a sample of 100,000 of the US population in the year 2000, we estimate that deaths from tobacco-related diseases at ages 35–84 occurring by the year 2050 would reduce by 249, equivalent to almost 700,000 in the whole US population. These estimates assume that the prevalence of current ZYN use in 2050 would be about a quarter of the total current use of cigarettes and ZYN. Sensitivity analyses varying the parameter values assumed in the main analyses confirm that the introduction of ZYN is likely to substantially decrease product-related deaths even under our least optimistic assumptions. For those smokers not intending to quit smoking, switching to ZYN can substantially reduce the risk of product-related deaths.

## Supplementary Information

**Additional file 1. **Population data for the US for the year 2000.

**Additional file 2. **Data on migration and births for the US for the years 2001-2049

**Additional file 3. **Base case transition probabilities.

**Additional file 4. **Data on mortality for US.

## Data Availability

All data used in this study are included in this published article and its supplementary information files, and the publicly available databases from which they came are referenced in the paper. The program used to carry out the analyses, written in R, is available from the authors on request.

## Abbreviations

CISNET: Cancer Intervention and Surveillance Modelling Network
NHIS: National Health Interview Survey

## References

[1] US Surgeon General. The health consequences of smoking - 50 years of progress: a report of the Surgeon General. Atlanta, Georgia: US Department of Health and Human Services, Centers for Disease Control and Prevention, National Center for Chronic Disease Prevention and Health Promotion, Office on Smoking and Health; 2014. Available: http://www.surgeongeneral.gov/library/reports/index.html.

[2] Royal College of Physicians. Nicotine without smoke: tobacco harm reduction. London: RCP; 2016. Available: www.rcplondon.ac.uk/projects/outputs/nicotine-without-smoke-tobacco-harm-reduction.

[3] Kozlowski LT, Abrams DB (2016). Obsolete tobacco control themes can be hazardous to public health: the need for updating views on absolute product risks and harm reduction. BMC Public Health.

[4] Abrams DB, Glasser AM, Villanti AC, Pearson JL, Rose S, Niaura RS (2018). Managing nicotine without smoke to save lives now: Evidence for harm minimization. Prev Med.

[5] Lee PN (2011). Summary of the epidemiological evidence relating snus to health. Regul Toxicol Pharmacol.

[6] McNeill A, Brose LS, Calder R, Bauld L, Robson D. Evidence review of e-cigarettes and heated tobacco products. A report commissioned by Public Health England. London: Public Health England; 2018. Available: https://www.gov.uk/government/uploads/system/uploads/attachment_data/file/684963/Evidence_review_of_e-cigarettes_and_heated_tobacco_products_2018.pdf.

[7] Schaller JP, Keller D, Poget L, Pratte P, Kaelin E, McHugh D (2016). Evaluation of the Tobacco Heating System 2.2. Part 2: Chemical composition, genotoxicity, cytotoxicity, and physical properties of the aerosol. Regul Toxicol Pharmacol.

[8] Mallock N, Böss L, Burk R, Danziger M, Welsch T, Hahn H (2018). Levels of selected analytes in the emissions of "heat not burn" tobacco products that are relevant to assess human health risks. Arch Toxicol.

[9] Lee PN (2013). Epidemiological evidence relating snus to health - an updated review based on recent publications. Harm Reduct J.

[10] Ramström L, Wikmans T (2014). Mortality attributable to tobacco among men in Sweden and other European countries: an analysis of data in a WHO report. Tob Induc Dis.

[11] Azzopardi D, Liu C, Murphy J. Chemical characterization of tobacco-free "modern" oral nicotine pouches and their position on the toxicant and risk continuums. Drug Chem Toxicol 2021;Published online ahead of print (10.1080/01480545.2021.1925691):1-9.(Epub 20210525) 10.1080/01480545.2021.1925691 34034614

[12] Lee PN, Abrams D, Bachand A, Baker G, Black R, Camacho O (2021). Estimating the population health impact of recently introduced modified risk tobacco products: a comparison of different approaches. Nicotine Tob Res.

[13] Vugrin ED, Rostron BL, Verzi SJ, Brodsky NS, Brown TJ, Choiniere CJ (2015). Modeling the potential effects of new tobacco products and policies: a dynamic population model for multiple product use and harm. PLoS One.

[14] Apelberg BJ, Feirman SP, Salazar E, Corey CG, Ambrose BK, Paredes A (2018). Potential public health effects of reducing nicotine levels in cigarettes in the United States. N Engl J Med.

[15] Lee PN, Fry JS, Hamling JF, Sponsiello-Wang Z, Baker G, Weitkunat R (2017). Estimating the effect of differing assumptions on the population health impact of introducing a Reduced Risk Tobacco Product in the USA. Regul Toxicol Pharmacol.

[16] Djurdjevic S, Lee PN, Weitkunat R, Sponsiello-Wang Z, Ludicke F, Baker G (2018). Modeling the population health impact of introducing a modified risk tobacco product into the U.S. market. Healthcare (Basel).

[17] Lee PN, Djurdjevic S, Weitkunat R, Baker G (2018). Estimating the population health impact of introducing a reduced-risk tobacco product into Japan. The effect of differing assumptions, and some comparisons with the U.S. Regul Toxicol Pharmacol.

[18] Rytsar R, Djurdjevic S, Nussbaum AK, Kaul A, Bennewitz E, Lee PN, et al. Estimated public health gains from German smokers switching to risk-reduced alternatives: Results from population health impact modelling. Res Sq 2020;Preprint: Dec 28, 2020. 10.21203/rs.3.rs-135255/v1.

[19] Human Mortality Database. University of California, BU and Max Planck Institute for Demographic Research (Germany). 2020. https://www.mortality.org/. Accessed 5 May.

[20] US Census Bureau. National Intercensal Datasets: 2000-2010. 2020. https://www.census.gov/data/datasets/time-series/demo/popest/intercensal-2000-2010-national.html.

[21] Freedman KS, Nelson NM, Feldman LL (2012). Smoking initiation among young adults in the United States and Canada, 1998–2010: a systematic review. Prev Chronic Dis.

[22] Centers for Disease Control Prevention (2008). Smoking-attributable mortality, years of potential life lost, and productivity losses - United States, 2000–2004. MMWR Morb Mortal Wkly Rep.

[23] Lee RD, Carter LR (1992). Modeling and forecasting U.S. Mortality. J Am Stat Assoc.

[24] Hill A, Camacho OM (2017). A system dynamics modelling approach to assess the impact of launching a new nicotine product on population health outcomes. Regul Toxicol Pharmacol.

[25] Nutt DJ, Phillips LD, Balfour D, Curran HV, Dockrell M, Foulds J (2014). Estimating the harms of nicotine-containing products using the MCDA approach. Eur Addict Res.

[26] Foxon F, Selya AS (2020). Electronic cigarettes, nicotine use trends and use initiation ages among US adolescents from 1999 to 2018. Addiction.

[27] Haugen SM. Almost twice as many daily users of snus as daily smokers. Statistik sentralbyra. Statistics Norway. 2022. https://www.ssb.no/en/helse/helseforhold-og-levevaner/statistikk/royk-alkohol-og-andre-rusmidler/tobacco-alcohol-and-other-drugs/almost-twice-as-many-daily-users-of-snus-as-daily-smokers.

[28] Kantar Health. Swedish Match. ZYN® User Profile. Study Report SMNA 20–003 Kantar Health; 2021.

[29] Tam J, Day HR, Rostron BL, Apelberg BJ (2015). A systematic review of transitions between cigarette and smokeless tobacco product use in the United States. BMC Public Health.

[30] Jackson RA, Ren C, Coleman B, Day HR, Chang CM, Kofie J, et al. Transitions to smokeless tobacco use among adult cigarette smokers in the Population Assessment of Tobacco and Health (PATH) Study, Waves 3–5 (2015–2019). Tob Control 2021;Published online ahead of print Dec 22, 2021. (Epub 20211222): 10.1136/tobaccocontrol-2021-056907. 10.1136/tobaccocontrol-2021-056907 PMC9213562 34937805

[31] Federal Trade Commission. Federal Trade Commission Smokess Tobacco Report for 2020. 2021. Available: http://www.ftc.gov/system/files/documents/reports/federal-trade-commission-cigarette-report-2020-smokeless-tobacco-report-2020/p114508fy20smokelesstobacco.pdf.

[32] Marynak KL, Wang X, Borowiecki M, Kim Y, Tynan MA, Emery S (2021). Nicotine pouch unit sales in the US, 2016–2020. JAMA.

[33] Levy DT, Borland R, Villanti AC, Niaura R, Yuan Z, Zhang Y (2017). The application of a decision-theoretic model to estimate the public health impact of vaporized nicotine product initiation in the United States. Nicotine Tob Res.

