# Supplementary material for: Estimating the public health impact had tobacco-free nicotine pouches been introduced into the US in 2000
Source: BMC Public Health. 2022 May 21;22:1025. doi: 10.1186/s12889-022-13441-0 (PMC9123784; doi:10.1186/s12889-022-13441-0)
Supplement: Supplementary file 1 — Additional file 1. Population data for the US for the year 2000. [file 12889_2022_13441_MOESM1_ESM.docx]

**Estimating the public health impact from introducing tobacco-free nicotine pouches into the US**

**Short title:** Public health gains from introducing tobacco-free nicotine pouches

Peter N Lee^1*^, John S Fry^2^, Tryggve Ljung^3^

^1^ P.N.Lee Statistics and Computing Ltd., 17 Cedar Road, Sutton, Surrey SM2 5DA, UK

^2^ RoeLee Statistics Ltd., 17 Cedar Road, Sutton, Surrey SM2 5DA, UK

^3^ Swedish Match., Sveavägen 44 8th Floor, SE-118 85 Stockholm, Sweden

**ADDITIONAL FILE 1 – Population data for the US for the year 2000**

# A1.1. Distribution (%) of age within sex

A1.2. Distribution (%) of current and former smoking by age group within sex (age 18+)

A1.3. Distribution (%) of current smoking by age group within sex (age 0-17)

A1.4. Distribution (%) of years quit in former smokers within age group and sex

# Table A1.1. Distribution (%) of age within sex

| Age | Male | Female | Age | Male | Female |
| --- | --- | --- | --- | --- | --- |
|  |  |  |  |  |  |
| 0 | 1.43 | 1.31 | 51 | 1.29 | 1.30 |
| 1 | 1.41 | 1.30 | 52 | 1.34 | 1.34 |
| 2 | 1.41 | 1.30 | 53 | 1.17 | 1.18 |
| 3 | 1.42 | 1.31 | 54 | 1.02 | 1.03 |
| 4 | 1.46 | 1.34 | 55 | 1.01 | 1.03 |
| 5 | 1.48 | 1.36 | 56 | 1.02 | 1.04 |
| 6 | 1.50 | 1.38 | 57 | 0.96 | 0.99 |
| 7 | 1.54 | 1.41 | 58 | 0.87 | 0.91 |
| 8 | 1.55 | 1.43 | 59 | 0.82 | 0.86 |
| 9 | 1.58 | 1.45 | 60 | 0.81 | 0.85 |
| 10 | 1.58 | 1.45 | 61 | 0.77 | 0.81 |
| 11 | 1.53 | 1.40 | 62 | 0.74 | 0.78 |
| 12 | 1.51 | 1.38 | 63 | 0.71 | 0.76 |
| 13 | 1.50 | 1.37 | 64 | 0.70 | 0.76 |
| 14 | 1.50 | 1.38 | 65 | 0.67 | 0.74 |
| 15 | 1.50 | 1.36 | 66 | 0.64 | 0.71 |
| 16 | 1.50 | 1.36 | 67 | 0.64 | 0.72 |
| 17 | 1.52 | 1.37 | 68 | 0.63 | 0.72 |
| 18 | 1.51 | 1.37 | 69 | 0.62 | 0.72 |
| 19 | 1.53 | 1.41 | 70 | 0.61 | 0.73 |
| 20 | 1.49 | 1.37 | 71 | 0.59 | 0.70 |
| 21 | 1.43 | 1.31 | 72 | 0.57 | 0.69 |
| 22 | 1.39 | 1.28 | 73 | 0.55 | 0.68 |
| 23 | 1.35 | 1.25 | 74 | 0.53 | 0.68 |
| 24 | 1.35 | 1.25 | 75 | 0.50 | 0.67 |
| 25 | 1.37 | 1.28 | 76 | 0.47 | 0.64 |
| 26 | 1.35 | 1.27 | 77 | 0.45 | 0.61 |
| 27 | 1.41 | 1.33 | 78 | 0.42 | 0.59 |
| 28 | 1.48 | 1.41 | 79 | 0.37 | 0.55 |
| 29 | 1.53 | 1.46 | 80 | 0.31 | 0.48 |
| 30 | 1.56 | 1.47 | 81 | 0.29 | 0.46 |
| 31 | 1.47 | 1.39 | 82 | 0.25 | 0.42 |
| 32 | 1.47 | 1.40 | 83 | 0.22 | 0.38 |
| 33 | 1.48 | 1.42 | 84 | 0.19 | 0.35 |
| 34 | 1.55 | 1.49 | 85 | 0.17 | 0.33 |
| 35 | 1.65 | 1.58 | 86 | 0.14 | 0.29 |
| 36 | 1.65 | 1.59 | 87 | 0.12 | 0.26 |
| 37 | 1.65 | 1.60 | 88 | 0.09 | 0.22 |
| 38 | 1.64 | 1.60 | 89 | 0.08 | 0.19 |
| 39 | 1.67 | 1.63 | 90 | 0.06 | 0.16 |
| 40 | 1.70 | 1.64 | 91 | 0.05 | 0.14 |
| 41 | 1.62 | 1.59 | 92 | 0.04 | 0.11 |
| 42 | 1.63 | 1.59 | 93 | 0.03 | 0.09 |
| 43 | 1.58 | 1.56 | 94 | 0.02 | 0.07 |
| 44 | 1.54 | 1.52 | 95 | 0.01 | 0.05 |
| 45 | 1.54 | 1.52 | 96 | 0.01 | 0.04 |
| 46 | 1.46 | 1.45 | 97 | 0.01 | 0.03 |
| 47 | 1.42 | 1.42 | 98 | 0.00 | 0.02 |
| 48 | 1.36 | 1.36 | 99 | 0.00 | 0.01 |
| 49 | 1.35 | 1.35 | 100 | 0.00 | 0.01 |
| 50 | 1.35 | 1.35 |  |  |  |
|  | |  | |  | |

# Table A1.2. Distribution (%) of current and former smoking by age group within sex (age 18+)

|  | Males | |  | Females | |
| --- | --- | --- | --- | --- | --- |
| Age group | Current smokers | Former smokers |  | Current smokers | Former smokers |
|  |  |  |  |  |  |
| 18-24 | 28.50 | 7.40 |  | 25.10 | 8.00 |
| 25-34 | 29.00 | 12.30 |  | 22.50 | 10.90 |
| 35-44 | 30.20 | 16.50 |  | 26.20 | 17.10 |
| 45-54 | 28.80 | 28.90 |  | 22.20 | 23.10 |
| 55-64 | 22.60 | 45.00 |  | 20.80 | 28.30 |
| 65-74 | 13.70 | 53.90 |  | 12.20 | 28.80 |
| 75-84 | 5.70 | 58.70 |  | 6.90 | 25.10 |
| ≥85 | 3.80 | 58.30 |  | 3.80 | 19.30 |
|  |  |  |  |  |  |

# Table A1.3. Distribution (%) of current smoking by age group within sex (age 0-17)

|  | Current smokers | |
| --- | --- | --- |
| Age | Males | Females |
|  |  |  |
| 0 | 0 | 0 |
| 1 | 0 | 0 |
| 2 | 0 | 0 |
| 3 | 0 | 0 |
| 4 | 0 | 0 |
| 5 | 0 | 0 |
| 6 | 0 | 0 |
| 7 | 0.09 | 0 |
| 8 | 0.21 | 0.03 |
| 9 | 0.21 | 0.42 |
| 10 | 0.60 | 0.59 |
| 11 | 1.54 | 1.36 |
| 12 | 3.10 | 3.17 |
| 13 | 5.52 | 5.96 |
| 14 | 8.50 | 9.74 |
| 15 | 13.15 | 14.48 |
| 16 | 19.48 | 19.90 |
| 17 | 23.95 | 24.65 |
|  |  |  |

# Table A1.4. Distribution (%) of years quit in former smokers within age group and sex

|  | Age | Years quit in former smokers | | | | | | | |
| --- | --- | --- | --- | --- | --- | --- | --- | --- | --- |
| Sex | Group | 0-1 | 1-4 | 5-9 | 10-14 | 15-19 | 20-24 | 25-29 | 30-34 |
|  |  |  |  |  |  |  |  |  |  |
| Males | 18-24 | 0.0 | 88.6 | 11.4 |  |  |  |  |  |
|  | 25-35 | 0.0 | 58.7 | 24.0 | 15.0 | 2.3 |  |  |  |
|  | 35-44 | 0.0 | 31.9 | 18.1 | 19.9 | 13.8 | 14.2 | 2.1 |  |
|  | 45-54 | 0.0 | 18.5 | 13.4 | 16.4 | 15.1 | 16.2 | 11.5 | 7.9 |
|  | 55-64 | 0.0 | 16.7 | 10.5 | 14.5 | 14.3 | 13.5 | 12.1 | 8.9 |
|  | 65-74 | 0.0 | 9.7 | 8.3 | 11.0 | 7.9 | 13.9 | 8.2 | 15.4 |
|  | 75-84 | 0.0 | 6.7 | 7.3 | 8.5 | 9.5 | 14.1 | 9.6 | 12.8 |
|  | 85+ | 0.0 | 6.6 | 7.0 | 8.2 | 8.2 | 10.4 | 11.1 | 11.5 |
|  |  |  |  |  |  |  |  |  |  |
|  |  | 35-39 | 40-44 | 45-49 | 50-54 | 55-59 | 60-64 | 65-69 | 70+ |
|  |  |  |  |  |  |  |  |  |  |
|  | 18-24 |  |  |  |  |  |  |  |  |
|  | 25-35 |  |  |  |  |  |  |  |  |
|  | 35-44 |  |  |  |  |  |  |  |  |
|  | 45-54 | 1.0 |  |  |  |  |  |  |  |
|  | 55-64 | 5.0 | 4.4 | 0.1 |  |  |  |  |  |
|  | 65-74 | 7.9 | 10.9 | 4.7 | 1.9 | 0.2 |  |  |  |
|  | 75-84 | 6.3 | 12.9 | 4.2 | 6.2 | 0.2 | 1.5 | 0.2 |  |
|  | 85+ | 5.8 | 13.6 | 2.5 | 2.4 | 4.5 | 3.1 | 1.6 | 3.5 |
|  |  |  |  |  |  |  |  |  |  |
|  |  | 0-1 | 1-4 | 5-9 | 10-14 | 15-19 | 20-24 | 25-29 | 30-34 |
|  |  |  |  |  |  |  |  |  |  |
| Females | 18-24 | 0.1 | 92.6 | 7.4 |  |  |  |  |  |
|  | 25-35 | 0.0 | 60.1 | 24.6 | 13.6 | 1.7 |  |  |  |
|  | 35-44 | 0.0 | 27.8 | 18.2 | 21.9 | 16.4 | 13.4 | 2.3 |  |
|  | 45-54 | 0.0 | 22.5 | 11.5 | 13.5 | 18.0 | 15.3 | 10.9 | 8.0 |
|  | 55-64 | 0.0 | 19.0 | 9.9 | 13.2 | 13.7 | 13.4 | 9.4 | 11.5 |
|  | 65-74 | 0.0 | 13.2 | 14.0 | 13.5 | 11.6 | 11.1 | 9.3 | 10.9 |
|  | 75-84 | 0.0 | 9.6 | 8.8 | 11.3 | 10.8 | 15.0 | 8.5 | 11.6 |
|  | 85+ | 0.0 | 14.4 | 3.4 | 16.5 | 4.9 | 11.6 | 4.6 | 11.7 |
|  |  |  |  |  |  |  |  |  |  |
|  |  | 35-39 | 40-44 | 45-49 | 50-54 | 55-59 | 60-64 | 65-69 | 70+ |
|  |  |  |  |  |  |  |  |  |  |
|  | 18-24 |  |  |  |  |  |  |  |  |
|  | 25.35 |  |  |  |  |  |  |  |  |
|  | 35-44 |  |  |  |  |  |  |  |  |
|  | 45-54 | 0.3 |  |  |  |  |  |  |  |
|  | 55-64 | 5.7 | 3.4 | 0.8 |  |  |  |  |  |
|  | 65-74 | 5.0 | 6.0 | 1.9 | 3.5 |  |  |  |  |
|  | 75-84 | 6.4 | 7.6 | 2.8 | 4.1 | 1.6 | 1.9 | 0.0 |  |
|  | 85+ | 3.0 | 4.0 | 6.7 | 7.5 | 4.0 | 5.0 | 2.7 | 0.0 |
|  |  |  |  |  |  |  |  |  |  |
