# Supplementary material for: Estimating the public health impact had tobacco-free nicotine pouches been introduced into the US in 2000
Source: BMC Public Health. 2022 May 21;22:1025. doi: 10.1186/s12889-022-13441-0 (PMC9123784; doi:10.1186/s12889-022-13441-0)
Supplement: Supplementary file 2 — Additional file 2. Data on migration and births for the US for the years 2001-2049 [file 12889_2022_13441_MOESM2_ESM.docx]

**Estimating the public health impact from introducing tobacco-free nicotine pouches into the US**

**Short title:** Public health gains from introducing tobacco-free nicotine pouches

Peter N Lee^1*^, John S Fry^2^, Tryggve Ljung^3^

^1^ P.N.Lee Statistics and Computing Ltd., 17 Cedar Road, Sutton, Surrey SM2 5DA, UK

^2^ RoeLee Statistics Ltd., 17 Cedar Road, Sutton, Surrey SM2 5DA, UK

^3^ Swedish Match., Sveavägen 44 8th Floor, SE-118 85 Stockholm, Sweden

**ADDITIONAL FILE 2 – Data on migrations and births for the US for 2001-2049**

Table A2.1. Estimates of net migration (from US Census Bureau 2008 National Population Projections)

Table A2.2. Estimates of numbers of births (from US Census Bureau 2008 National Population Projections)

# Table A2.1. Estimates of net migration (from US Census Bureau 2008 National Population Projections)

| Year | Net Migration | Year | Net Migration |
| --- | --- | --- | --- |
|  |  |  |  |
| 2001 | 1,172,779 | 2026 | 1,587,656 |
| 2002 | 1,017,718 | 2027 | 1,606,748 |
| 2003 | 901,141 | 2028 | 1,625,881 |
| 2004 | 1,198,067 | 2029 | 1,645,011 |
| 2005 | 1,147,895 | 2030 | 1,664,183 |
| 2006 | 1,227,060 | 2031 | 1,683,268 |
| 2007 | 1,198,810 | 2032 | 1,702,405 |
| 2008 | 1,298,964 | 2033 | 1,721,545 |
| 2009 | 1,318,823 | 2034 | 1,740,695 |
| 2010 | 1,338,370 | 2035 | 1,759,827 |
| 2011 | 1,299,753 | 2036 | 1,778,907 |
| 2012 | 1,319,079 | 2037 | 1,798,046 |
| 2013 | 1,338,343 | 2038 | 1,817,202 |
| 2014 | 1,357,638 | 2039 | 1,836,308 |
| 2015 | 1,376,867 | 2040 | 1,855,450 |
| 2016 | 1,396,088 | 2041 | 1,874,562 |
| 2017 | 1,415,292 | 2042 | 1,893,704 |
| 2018 | 1,434,452 | 2043 | 1,912,798 |
| 2019 | 1,453,630 | 2044 | 1,931,968 |
| 2020 | 1,472,777 | 2045 | 1,951,077 |
| 2021 | 1,491,943 | 2046 | 1,970,214 |
| 2022 | 1,511,092 | 2047 | 1,989,330 |
| 2023 | 1,530,231 | 2048 | 2,008,445 |
| 2024 | 1,549,403 | 2049 | 2,027,607 |
| 2025 | 1,568,510 |  |  |
|  |  |  |  |

# Table A2.2. Estimates of numbers of births for 2001-2049 (from US Census Bureau 2008 National Population Projections)

| Year | Births | Year | Births |
| --- | --- | --- | --- |
|  |  |  |  |
| 2001 | 3,997,261 | 2026 | 4,737,563 |
| 2002 | 3,996,506 | 2027 | 4,764,647 |
| 2003 | 4,022,456 | 2028 | 4,793,585 |
| 2004 | 4,067,803 | 2029 | 4,824,637 |
| 2005 | 4,094,564 | 2030 | 4,857,932 |
| 2006 | 4,120,248 | 2031 | 4,893,400 |
| 2007 | 4,150,692 | 2032 | 4,930,850 |
| 2008 | 4,186,384 | 2033 | 4,970,038 |
| 2009 | 4,226,315 | 2034 | 5,010,645 |
| 2010 | 4,268,440 | 2035 | 5,052,322 |
| 2011 | 4,310,502 | 2036 | 5,094,732 |
| 2012 | 4,350,781 | 2037 | 5,137,505 |
| 2013 | 4,388,472 | 2038 | 5,180,353 |
| 2014 | 4,423,183 | 2039 | 5,222,983 |
| 2015 | 4,454,996 | 2040 | 5,265,209 |
| 2016 | 4,484,444 | 2041 | 5,306,882 |
| 2017 | 4,512,345 | 2042 | 5,347,893 |
| 2018 | 4,539,134 | 2043 | 5,388,200 |
| 2019 | 4,565,053 | 2044 | 5,427,768 |
| 2020 | 4,590,367 | 2045 | 5,466,633 |
| 2021 | 4,615,146 | 2046 | 5,504,818 |
| 2022 | 4,639,429 | 2047 | 5,542,416 |
| 2023 | 4,663,383 | 2048 | 5,579,488 |
| 2024 | 4,687,407 | 2049 | 5,616,140 |
| 2025 | 4,711,967 |  |  |
|  |  |  |  |
