# Supplementary material for: Estimating the public health impact had tobacco-free nicotine pouches been introduced into the US in 2000
Source: BMC Public Health. 2022 May 21;22:1025. doi: 10.1186/s12889-022-13441-0 (PMC9123784; doi:10.1186/s12889-022-13441-0)
Supplement: Supplementary file 3 — Additional file 3. Base case transition probabilities. [file 12889_2022_13441_MOESM3_ESM.docx]

**Estimating the public health impact from introducing tobacco-free nicotine pouches into the US**

**Short title:** Public health gains from introducing tobacco-free nicotine pouches

Peter N Lee^1*^, John S Fry^2^, Tryggve Ljung^3^

^1^ P.N.Lee Statistics and Computing Ltd., 17 Cedar Road, Sutton, Surrey SM2 5DA, UK

^2^ RoeLee Statistics Ltd., 17 Cedar Road, Sutton, Surrey SM2 5DA, UK

^3^ Swedish Match., Sveavägen 44 8th Floor, SE-118 85 Stockholm, Sweden

**ADDITIONAL FILE 3 – Base case transition probabilities**

Yearly transition probabilities (%) in the Base Case (Table A3.1) and details of the methodology used to derive them.

**Methodology**

The sex- and age-specific cigarette initiation and cessation rates used for the Base Case were derived from US birth cohort smoking history data. The estimates were generated as part of the CISNET (Cancer Intervention and Surveillance Modeling Network) Lung Working Group, sponsored by the National Cancer Institute, and have been used to model and quantify the effect of reduced cigarette smoking on lung cancer mortality in the US from 1975 to 2000 [1]. Researchers from the Working Group estimated cohort smoking histories using 25 NHIS surveys administered from 1965 to 2001 [2]. They estimated current- and ever-smoking prevalence and initiation and cessation rates by age and sex for five-year birth cohorts of individuals born between 1900 and 1984. A detailed explanation of their methodological approach and the resulting data is available  [3].

We used cohort- and age-specific smoking initiation rates for the period closest to 2000, the initial year in our population model projections. We used initiation rates for ages 0-19 years from 1980-1984 birth cohorts, rates for ages 20-24 years from 1975-1979 cohorts, rates for ages 25-29 years from 1970-1974 cohorts, and rates for age 30 years from 1965-1969 cohorts. We did not include cigarette initiation beyond the age of 30 in our model, given that the overwhelming majority of smoking initiation has occurred by this age [4]. Table A3.1 shows the estimated initiation rates by sex and age used in our modelling. The rates reflect initiation to established use as indicated by NHIS participants reporting if they had smoked at least 100 cigarettes in their lives. These age-specific initiation rates are assumed to remain constant throughout the projection period in the Base Case.

We also used smoking cessation rates in the model that came from CISNET cohort data. Rates for ages 1-18 years came from 1980-1984 birth cohorts, rates for ages 19-24 years from 1975-1979 cohorts, rates for ages 25-29 years from 1970-1974 cohorts, and so on through ages 83-85 from 1900-1904 cohorts. Cessation rates are available from the cohort data through age 85 for males and age 84 for females. Beyond these ages, cessation rates in the model are assumed to remain equal to the last available estimate from the cohort data. Table A3.1 also shows the estimated cessation rates by sex and age that are used in our modelling. The modelling methodology allows for relapse, but transition probabilities for relapsing are set to zero in the modelling simulations presented here because the CISNET cessation rates reflect successful smoking cessation for at least two years. These age-specific cessation rates are assumed to remain constant throughout the projection period in the Base Case.

**References**

1. Moolgavkar SH, Holford TR, Levy DT, Kong CY, Foy M, Clarke L, et al. Impact of reduced tobacco smoking on lung cancer mortality in the United States during 1975-2000. J Natl Cancer Inst 2012;104(7):541-8.

2. Anderson CM, Burns DM, Dodd KW, Feuer EJ. The Impact of the Reduction in Tobacco Smoking on U.S. Lung Cancer Mortality (1975-2000): Collective Results from the Cancer Intervention and Surveillance Modeling Network (CISNET). Section I. Model inputs. Chapter 2: Birth-cohort-specific estimates of smoking behaviors for the U.S. population. Risk Anal 2012;32(s1):S14-S24. doi:10.1111/j.1539-6924.2011.01703.x.

3. Feuer EJ, Levy DT, McCarthy WJ. The Impact of the Reduction in Tobacco Smoking on U.S. Lung Cancer Mortality (1975-2000): Collective Results from the Cancer Intervention and Surveillance Modeling Network (CISNET). Perspective. Chapter 1: The impact of the reduction in tobacco smoking on U.S. lung cancer mortality, 1975-2000: An introduction to the problem. Risk Anal 2012;32(s1):S6-S13. doi:10.1111/j.1539-6924.2011.01745.x.

4. Freedman KS, Nelson NM, Feldman LL. Smoking initiation among young adults in the United States and Canada, 1998-2010: a systematic review. Prev Chronic Dis 2012;9:E05.

# Table A3.1. Yearly transition probabilities (%) in the Base Case

|  | Yearly transition probabilities in the Base Case | | | | |
| --- | --- | --- | --- | --- | --- |
|  | Males | |  | Females | |
| Age | Initiation | Cessation |  | Initiation | Cessation |
|  |  |  |  |  |  |
| 0-7 | 0.00 | 0.00 |  | 0.00 | 0.00 |
| 8 | 0.06 | 0.00 |  | 0.12 | 0.00 |
| 9 | 0.08 | 0.00 |  | 0.21 | 0.00 |
| 10 | 0.37 | 0.00 |  | 0.31 | 0.00 |
| 11 | 0.92 | 0.00 |  | 0.78 | 0.00 |
| 12 | 1.64 | 0.00 |  | 1.79 | 0.02 |
| 13 | 2.33 | 0.19 |  | 2.87 | 0.27 |
| 14 | 3.36 | 0.56 |  | 4.07 | 0.59 |
| 15 | 5.40 | 0.99 |  | 5.56 | 0.96 |
| 16 | 7.01 | 1.45 |  | 6.82 | 1.37 |
| 17 | 6.75 | 1.92 |  | 6.22 | 1.80 |
| 18 | 5.14 | 2.38 |  | 4.47 | 2.23 |
| 19 | 2.42 | 1.89 |  | 1.87 | 2.66 |
| 20 | 3.22 | 2.35 |  | 2.23 | 3.24 |
| 21 | 1.99 | 2.82 |  | 1.71 | 3.83 |
| 22 | 1.08 | 3.28 |  | 1.04 | 4.42 |
| 23 | 0.51 | 3.75 |  | 0.54 | 5.02 |
| 24 | 0.32 | 4.21 |  | 0.14 | 5.61 |
| 25 | 0.61 | 3.29 |  | 0.36 | 3.48 |
| 26 | 0.43 | 3.52 |  | 0.29 | 3.68 |
| 27 | 0.30 | 3.75 |  | 0.25 | 3.88 |
| 28 | 0.17 | 3.97 |  | 0.16 | 4.08 |
| 29 | 0.03 | 4.20 |  | 0.02 | 4.28 |
| 30 | 0.23 | 3.60 |  | 0.21 | 2.82 |
| 31 | 0.00 | 3.94 |  | 0.00 | 2.88 |
| 32 | 0.00 | 4.27 |  | 0.00 | 2.95 |
| 33 | 0.00 | 4.60 |  | 0.00 | 3.01 |
| 34 | 0.00 | 4.93 |  | 0.00 | 3.07 |
| 35 | 0.00 | 2.72 |  | 0.00 | 2.64 |
| 36 | 0.00 | 2.78 |  | 0.00 | 2.66 |
| 37 | 0.00 | 2.85 |  | 0.00 | 2.68 |
| 38 | 0.00 | 2.91 |  | 0.00 | 2.69 |
| 39 | 0.00 | 2.97 |  | 0.00 | 2.71 |
| 40 | 0.00 | 2.71 |  | 0.00 | 2.36 |
| 41 | 0.00 | 2.74 |  | 0.00 | 2.33 |
| 42 | 0.00 | 2.77 |  | 0.00 | 2.31 |
| 43 | 0.00 | 2.80 |  | 0.00 | 2.28 |
| 44 | 0.00 | 2.83 |  | 0.00 | 2.26 |
| 45 | 0.00 | 2.50 |  | 0.00 | 3.00 |
| 46 | 0.00 | 2.50 |  | 0.00 | 3.05 |
| 47 | 0.00 | 2.49 |  | 0.00 | 3.10 |
| 48 | 0.00 | 2.48 |  | 0.00 | 3.15 |
| 49 | 0.00 | 2.48 |  | 0.00 | 3.20 |
| 50 | 0.00 | 3.18 |  | 0.00 | 3.41 |
| 51 | 0.00 | 3.21 |  | 0.00 | 3.52 |
| 52 | 0.00 | 3.23 |  | 0.00 | 3.63 |
| 53 | 0.00 | 3.26 |  | 0.00 | 3.74 |
| 54 | 0.00 | 3.57 |  | 0.00 | 3.85 |
| 55 | 0.00 | 3.61 |  | 0.00 | 3.62 |
| 56 | 0.00 | 3.65 |  | 0.00 | 3.72 |
| 57 | 0.00 | 3.69 |  | 0.00 | 3.82 |
| 58 | 0.00 | 3.73 |  | 0.00 | 3.93 |
| 59 | 0.00 | 3.77 |  | 0.00 | 4.46 |
| 60 | 0.00 | 4.20 |  | 0.00 | 4.61 |
| 61 | 0.00 | 4.26 |  | 0.00 | 4.77 |
| 62 | 0.00 | 4.32 |  | 0.00 | 4.92 |
| 63 | 0.00 | 4.38 |  | 0.00 | 5.08 |
| 64 | 0.00 | 4.44 |  | 0.00 | 4.75 |
| 65 | 0.00 | 5.44 |  | 0.00 | 4.91 |
| 66 | 0.00 | 5.58 |  | 0.00 | 5.07 |
| 67 | 0.00 | 5.72 |  | 0.00 | 5.23 |
| 68 | 0.00 | 5.86 |  | 0.00 | 5.39 |
| 69 | 0.00 | 6.93 |  | 0.00 | 5.55 |
| 70 | 0.00 | 7.15 |  | 0.00 | 6.23 |
| 71 | 0.00 | 7.36 |  | 0.00 | 6.45 |
| 72 | 0.00 | 7.58 |  | 0.00 | 6.67 |
| 73 | 0.00 | 7.79 |  | 0.00 | 6.89 |
| 74 | 0.00 | 6.92 |  | 0.00 | 5.96 |
| 75 | 0.00 | 7.09 |  | 0.00 | 6.14 |
| 76 | 0.00 | 7.27 |  | 0.00 | 6.33 |
| 77 | 0.00 | 7.45 |  | 0.00 | 6.52 |
| 78 | 0.00 | 7.84 |  | 0.00 | 6.03 |
| 79 | 0.00 | 8.02 |  | 0.00 | 6.19 |
| 80 | 0.00 | 8.20 |  | 0.00 | 6.35 |
| 81 | 0.00 | 8.38 |  | 0.00 | 6.51 |
| 82 | 0.00 | 7.54 |  | 0.00 | 6.75 |
| 83 | 0.00 | 7.73 |  | 0.00 | 6.96 |
| 84 | 0.00 | 7.93 |  | 0.00 | 7.18 |
|  |  |  |  |  |  |
