# Supplementary material for: Estimating the public health impact had tobacco-free nicotine pouches been introduced into the US in 2000
Source: BMC Public Health. 2022 May 21;22:1025. doi: 10.1186/s12889-022-13441-0 (PMC9123784; doi:10.1186/s12889-022-13441-0)
Supplement: Supplementary file 4 — Additional file 4. Data on mortality for US. [file 12889_2022_13441_MOESM4_ESM.docx]

**Estimating the public health impact from introducing tobacco-free nicotine pouches into the US**

**Short title:** Public health gains from introducing tobacco-free nicotine pouches

Peter N Lee^1*^, John S Fry^2^, Tryggve Ljung^3^

^1^ P.N.Lee Statistics and Computing Ltd., 17 Cedar Road, Sutton, Surrey SM2 5DA, UK

^2^ RoeLee Statistics Ltd., 17 Cedar Road, Sutton, Surrey SM2 5DA, UK

^3^ Swedish Match., Sveavägen 44 8th Floor, SE-118 85 Stockholm, Sweden

**ADDITIONAL FILE 4 – Data on mortality for US**

Table A4.1. Annual probability (%) of dying for never smokers (ages 0-34 years)

Table A4.2. Mortality scaling factors for males

Table A4.3. Mortality scaling factors for females

Table A4.4. Annual probability (%) of dying for never smokers (ages ≥35 years)

Table A4.5. Relative risk of dying for current smokers compared to never smokers (ages ≥35 years)

#

# Table A4.1. Annual probability (%) of dying for never smokers (ages 0-34 years)

|  | Annual probability of dying for never users (ages 0-34) | |
| --- | --- | --- |
| Age | Males | Females |
|  |  |  |
| 0 | 0.788 | 0.648 |
| 1 | 0.056 | 0.046 |
| 2 | 0.038 | 0.031 |
| 3 | 0.029 | 0.020 |
| 4 | 0.022 | 0.019 |
| 5 | 0.021 | 0.016 |
| 6 | 0.018 | 0.014 |
| 7 | 0.017 | 0.015 |
| 8 | 0.016 | 0.013 |
| 9 | 0.016 | 0.013 |
| 10 | 0.018 | 0.012 |
| 11 | 0.021 | 0.015 |
| 12 | 0.022 | 0.014 |
| 13 | 0.026 | 0.017 |
| 14 | 0.035 | 0.022 |
| 15 | 0.049 | 0.027 |
| 16 | 0.072 | 0.037 |
| 17 | 0.093 | 0.040 |
| 18 | 0.116 | 0.047 |
| 19 | 0.132 | 0.045 |
| 20 | 0.135 | 0.046 |
| 21 | 0.142 | 0.048 |
| 22 | 0.137 | 0.047 |
| 23 | 0.134 | 0.047 |
| 24 | 0.135 | 0.046 |
| 25 | 0.127 | 0.049 |
| 26 | 0.127 | 0.050 |
| 27 | 0.130 | 0.053 |
| 28 | 0.129 | 0.055 |
| 29 | 0.134 | 0.058 |
| 30 | 0.129 | 0.060 |
| 31 | 0.140 | 0.067 |
| 32 | 0.145 | 0.073 |
| 33 | 0.156 | 0.080 |
| 34 | 0.169 | 0.086 |
|  |  |  |

# Table A4.2. Mortality scaling factors for males

|  | Scaling factors applied to mortality risks (Males) | | | | | |
| --- | --- | --- | --- | --- | --- | --- |
| Age/Year | 2000 | 2010 | 2020 | 2030 | 2040 | 2050 |
|  |  |  |  |  |  |  |
| 0 | 1 | 0.719 | 0.516 | 0.371 | 0.267 | 0.192 |
| 1 | 1 | 0.769 | 0.592 | 0.455 | 0.350 | 0.269 |
| 5 | 1 | 0.774 | 0.599 | 0.463 | 0.359 | 0.278 |
| 10 | 1 | 0.830 | 0.689 | 0.572 | 0.475 | 0.394 |
| 15 | 1 | 0.942 | 0.887 | 0.835 | 0.786 | 0.740 |
| 20 | 1 | 0.934 | 0.872 | 0.814 | 0.760 | 0.709 |
| 25 | 1 | 0.957 | 0.917 | 0.877 | 0.840 | 0.804 |
| 30 | 1 | 0.972 | 0.944 | 0.917 | 0.891 | 0.866 |
| 35 | 1.024 | 0.966 | 0.911 | 0.859 | 0.810 | 0.764 |
| 40 | 1.043 | 0.939 | 0.845 | 0.761 | 0.685 | 0.617 |
| 45 | 1.056 | 0.921 | 0.803 | 0.701 | 0.611 | 0.533 |
| 50 | 1.064 | 0.911 | 0.780 | 0.668 | 0.572 | 0.490 |
| 55 | 1.058 | 0.919 | 0.797 | 0.692 | 0.601 | 0.521 |
| 60 | 1.053 | 0.925 | 0.813 | 0.714 | 0.627 | 0.551 |
| 65 | 1.048 | 0.933 | 0.831 | 0.740 | 0.659 | 0.586 |
| 70 | 1.039 | 0.944 | 0.857 | 0.778 | 0.706 | 0.641 |
| 75 | 1.037 | 0.947 | 0.865 | 0.790 | 0.722 | 0.660 |
| 80 | 1.029 | 0.958 | 0.893 | 0.832 | 0.775 | 0.722 |
| 85+ | 1.017 | 0.976 | 0.936 | 0.899 | 0.863 | 0.828 |
|  |  |  |  |  |  |  |

# Table A4.3. Mortality scaling factors for females

|  | Scaling factors applied to mortality risks (Females) | | | | | |
| --- | --- | --- | --- | --- | --- | --- |
| Age/Year | 2000 | 2010 | 2020 | 2030 | 2040 | 2050 |
|  |  |  |  |  |  |  |
| 0 | 1 | 0.752 | 0.565 | 0.425 | 0.320 | 0.240 |
| 1 | 1 | 0.779 | 0.607 | 0.473 | 0.369 | 0.288 |
| 5 | 1 | 0.800 | 0.640 | 0.512 | 0.409 | 0.327 |
| 10 | 1 | 0.851 | 0.725 | 0.617 | 0.525 | 0.447 |
| 15 | 1 | 0.921 | 0.848 | 0.781 | 0.719 | 0.662 |
| 20 | 1 | 0.895 | 0.800 | 0.716 | 0.641 | 0.573 |
| 25 | 1 | 0.886 | 0.785 | 0.695 | 0.616 | 0.545 |
| 30 | 1 | 0.878 | 0.771 | 0.677 | 0.595 | 0.522 |
| 35 | 1.057 | 0.920 | 0.801 | 0.697 | 0.606 | 0.528 |
| 40 | 1.060 | 0.916 | 0.791 | 0.683 | 0.590 | 0.509 |
| 45 | 1.057 | 0.921 | 0.802 | 0.699 | 0.609 | 0.530 |
| 50 | 1.051 | 0.928 | 0.819 | 0.722 | 0.637 | 0.562 |
| 55 | 1.041 | 0.942 | 0.852 | 0.771 | 0.698 | 0.631 |
| 60 | 1.039 | 0.945 | 0.860 | 0.782 | 0.712 | 0.648 |
| 65 | 1.041 | 0.942 | 0.853 | 0.772 | 0.699 | 0.633 |
| 70 | 1.044 | 0.937 | 0.841 | 0.754 | 0.677 | 0.607 |
| 75 | 1.052 | 0.927 | 0.818 | 0.721 | 0.636 | 0.561 |
| 80 | 1.045 | 0.936 | 0.839 | 0.752 | 0.673 | 0.603 |
| 85+ | 1.025 | 0.964 | 0.906 | 0.853 | 0.802 | 0.754 |
|  |  |  |  |  |  |  |

# Table A4.4. Annual probability (%) of dying for never smokers (ages ≥35 years)

|  | Annual probability of dying for never smokers (ages ≥35 years) | |
| --- | --- | --- |
| Age group | Males | Females |
|  |  |  |
| 35-44 | 0.18 | 0.10 |
| 45-54 | 0.37 | 0.23 |
| 55-64 | 0.64 | 0.52 |
| 65-74 | 1.72 | 1.21 |
| 75-84 | 4.67 | 3.57 |
| 85+ | 13.16 | 12.18 |
|  |  |  |

# Table A4.5. Relative risk of dying for current smokers compared to never smokers (ages ≥35 years)

|  | Relative risk of dying for current smokers compared to never smokers | |
| --- | --- | --- |
| Age group | Males | Females |
|  |  |  |
| 35-44 | 1.84 | 3.34 |
| 45-54 | 2.04 | 2.42 |
| 55-64 | 2.79 | 2.35 |
| 65-74 | 2.77 | 2.90 |
| 75-84 | 2.19 | 2.57 |
| 85+ | 1.36 | 1.56 |
|  |  |  |
